# A Novel Approach to the Facile Growth and Organization of Photothermal Prussian Blue Nanocrystals on Different Surfaces

**DOI:** 10.3390/nano11071749

**Published:** 2021-07-02

**Authors:** Giang Ngo, Gautier Félix, Christophe Dorandeu, Jean-Marie Devoisselle, Luca Costa, Pierre-Emmanuel Milhiet, Yannick Guari, Joulia Larionova, Joël Chopineau

**Affiliations:** 1 ICGM, Univ Montpellier, CNRS, ENSCM, 34090 Montpellier, France; honggiang91@gmail.com (G.N.); christophe.dorandeu@umontpellier.fr (C.D.); jean-marie.devoisselle@umontpellier.fr (J.-M.D.); joulia.larionova@umontpellier.fr (J.L.); 2 CBS, Univ Montpellier, CNRS, INSERM, 34090 Montpellier, France; costa@cbs.cnrs.fr (L.C.); pierre-emmanuel.milhiet@cbs.cnrs.fr (P.-E.M.)

**Keywords:** Prussian blue, nanostructures, surface growth, atomic force microscopy, gold surface, supported lipid bilayers, films

## Abstract

We report here a novel “one-pot” approach for the controlled growth and organization of Prussian blue nanostructures on three different surfaces: pure Au^0^, cysteamine-functionalized Au^0^, and SiO_2_-supported lipid bilayers with different natures of lipids. We demonstrate that fine control over the size, morphology, and the degree and homogeneity of the surface coverage by Prussian Blue (PB) nanostructures may be achieved by manipulating different parameters, which are the precursor concentration, the nature of the functional groups or the nature of lipids on the surfaces. This allows the growth of isolated PB nanopyramids and nanocubes or the design of thin dense films over centimeter square surfaces. The formation of unusual Prussian blue nanopyramids is discussed. Finally, we demonstrate, by using experimental techniques and theoretical modeling, that PB nanoparticles deposited on the gold surface exhibit strong photothermal properties, permitting a rapid temperature increase up to 90 °C with a conversion of the laser power of almost 50% for power source heat.

## 1. Introduction

The famous cyano-bridged coordination polymer well known under the name Prussian Blue (PB) was discovered in 1706 by J. C. Dippel and J. J. von Diesbach as a deep blue pigment [1]. Its crystal structure and fascinating chemical and physical properties have been investigated during the 300 years after its invention, but it remains an exciting object of modern research [2]. PB has the chemical formula A_1−x_Fe^III^[Fe^II^(CN)_6_]_1−x/4_▯_x/4_, where A is an alkaline ion and ▯ represents a cyanometallate vacancy needed to ensure the electroneutrality. In its crystal structure, the Fe^2+^ and Fe^3+^ ions are linked through cyano-bridges to form a porous three-dimensional face-centered cubic (*fcc*) crystal structure of the NaCl type. This compound was the first in the large series of widely developed cyano-bridged coordination polymers, called Prussian Blue Analogous (PBAs), A_x_M[M′(CN)_6_]_y_▯_z_ (where M and M′ are transition metal ions). Due to their exciting magnetic, optical, and catalytic properties, mechanical robustness, good hydrothermal stability, porosity, and host–guest properties, PB and PBAs have widely been developed for different applications including electrochemical sensing [3,4], energy storage [5,6,7], gas storage and separation [8,9,10], biosensor construction, and others [11,12,13].

In the last two decades, PB and its analogues have been the subject of renewed interest linked with the development of these materials at the nano-scale. Pioneering works have been reported by S. P. Moulik and coll [14] and S. Mann and coll. [15], both devoted to the use of reverse micellar systems for the synthesis and stabilization of cubic nanoparticles of Cu_3_[Fe(CN)_6_]_2_-PBA and PB itself. Since then, research efforts in this field have led to the development of different approaches for the synthesis of PB(A)s nanoparticles in solution with various size ranging from a few to several hundred nanometres [6,16,17,18,19,20]. These efforts have been linked not only with the purely fundamental interest of how the size and morphology impact the physical and chemical properties of these nano-objects, but also due to a wide range of their potential applications including electronics, sensors [21,22], biology, and health [23,24].

A specific interest in the field of PB and PBA nanostructures, which open a tremendous perspective for the integration of these nano-objects in various devices for further applications, has also been focused on the organisation of nanocrystals on various surfaces or on the preparation of nanocrystalline thin films. Such investigations were first developed to elaborate modified electrodes using electrochemical deposition [25], in situ chemically modified graphite powder [26], mechanical attachment [27] or photochemical deposition [28] methods. However, concomitant to the development of PB and PBA nano-objects in colloidal solution, many studies have focused on the exploration of new routes for the formation of PBA-nanostructured thin films and to control their thickness and homogeneity [29]. The most investigated approach consists of the sequential growth of PBA layers by subsequent and alternate impregnation of selected functionalized surface by metal salts and cyanometallate precursors, which is usually named multiple sequential assembly (MSA) or layer-by-layer deposition [30,31,32,33,34]. Indeed, magnetic and photomagnetic PBA films have been obtained on polyethylene terephthalate polymer surfaces (Melinex) [35,36], or have been grown on a Si(100) surface functionalized by Ni(II) complexes acting as an anchoring site for nanoparticle growth. PBA thin films have also been obtained on a quartz or indium tin oxide surface [33]. Finally, we can also cite growth of nano-objects by nanopatterning on a gold surface [37]. This method has resulted in a relatively homogeneous surface of small crystalline nano-objects, implying the crystallization of PB(A)s based on the initial formation of nucleation sites. However, the formation of regular nanostructures on large surface areas and the fine control of their size and morphology are quite challenging considering the fast crystallization rate of PB(A)s. Moreover, this method is rather complex to enforce since it needs multiple treatment/washing cycles. Note also that this approach has mainly been developed for the formation of different PBA nanostructured films, such as NiCr, NiFe, NiCr-NiFe [34], CuMo [38], and CoFe [35,37] for the investigation of their magnetic and photomagnetic properties, and surprisingly, the formation of PB films on the surface has been reported only scarcely [39].

Herein, we report a simpler one-step approach based on the simultaneous addition of precursors, conducting controlled surface growth of isolated PB nanocrystals or their organization into thin films. We demonstrate the feasibility of this method by using three different surfaces: gold, functionalized gold, and glass-supported lipid bilayers (SLB) [40,41]. This method combines the advantages of a simple PB NP synthesis with the high PB surface coverage of the layer-by-layer deposition procedure, without the constraint of several centrifugation washing steps or the complex and a non-automatized sequential immersion process. Moreover, there is no restriction on conductive surfaces as the electrodeposition technique requires.

In this study, we focused on PB nanostructures because they present an important interest for their photothermal properties. Indeed, they exhibit strong absorption properties in the near-infrared region (NIR), with a high molar extinction coefficient due to the Fe^2+^ to Fe^3+^ charge transfer band situated between 650 and 850 nm and high light-to-heat conversion coefficient, indicating that these nanoparticles efficiently convert laser irradiation into thermal energy making them very interesting as new NIR-driven photothermal agents [42,43]. Moreover, PB nano-objects in aqueous solutions present a higher photothermal conversion efficiency than Au nanorods (41.4% vs. 21%) and an excellent photothermal stability compared to Au nano-rods and organic dyes used as conventional photothermal agents. On the other hand, the photothermal properties of PB nanoparticles have widely been investigated in solutions, and there are only a few reports devoted to the study of photothermal effects of PB nanoparticles deposited on the surface for antibacterial applications [44,45].

The choice of the gold surface in our work was motivated by its high affinity for the cyanide groups able to directly fix PB and PBA nano-objects [46], by its easy functionalization, and by its optical/plasmonic properties. Note that only limited numbers of published works addressed the deposition of PB on the gold surface despite an important interest of these nano-objects for applications. We can cite here the direct deposition of the PB nanoparticles on bare gold-covered electrodes [47,48], electrodeposition of an epitaxial PB film on a single-crystal Au(110) substrate [49], or by using the MSA method with patterning by a photo or electron beam lithography gold surface [39]. On the other hand, despite a great interest attracted by interactions between supported lipid bilayers (SLB) and nano-objects, they have never been considered until now as a supported surface for the in-situ growth of nanoparticles. The SLB can be obtained on various supports, such as silica, gold, titanium or polymer cushions using a variety of top-down approaches. They offer the possibility to have soft interfaces with interesting physico–chemical properties and the possibility to modulate the surface charge by using different lipids or their mixture [41,50,51] and are usually used as model membrane systems for the reconstitution/interactions of proteins and for the studies of nanoparticle interactions with membranes [50,52]. In this work, we took advantage of these different potentialities and investigate the in-situ growth of PB nano-objects on gold and SLB surfaces, demonstrating for the first time the possibility of direct nanoparticles growth on the latter.

Finally, we demonstrate, by using experimental technique and theoretical modeling, that PB nanoparticles deposited on the gold surface exhibit strong photothermal properties, permitting a rapid temperature increase up to 90 °C with a conversion of laser power of almost 50% in the power source heat.

## 2. Materials and Methods

### 2.1. Chemicals and Substrates

All chemical reagents were purchased and used without further purification: 2-aminoethanethiol hydrochloride (Sigma Aldrich, St. Quentin Fallavier, France, 98%), sodium ferrocyanide and Iron (III) chloride (Fluka), lipids 1-palmitoyl-2-oleoyl-sn-glycero-3-phosphocholine (POPC), and 1,2-dioleoyl-3-(trimethylammonium) propane (DOTAP) were purchased from Avanti Polar Lipids. Two types of planar substrates were used. The first substrate was gold-coated glass coverslips with a diameter of 14 mm; gold coating (including 2 ± 0.5 nm thickness of the chromium layer and 47 ± 3 nm thickness of the gold layer) was performed at the “Centrale de Technologie en Micro et nanoélectronique” of Montpellier University using the electron gun evaporator technique (Univex 250, Leybold, UK). The second substrate was QCM-D sensors (Biolin Scientific, Les Ulis, France) with a diameter of 14 mm and a total thickness of 0.3 mm. This surface was coated with 50 nm silica (SiO_2_) and was used to support lipid bilayers.

Three different types of surfaces were considered starting material; bare gold-coated glass, cysteamine-functionalized gold-coated glass, and SLB. A cysteamine-functionalized gold surface was obtained by immersing the gold-coated glass slide in an ethanol solution containing 5 mM of cysteamine hydrochloride for 15 h. Then, the surface was thoroughly rinsed with MilliQ water and neutralized by immersing in 35 mL of NaOH solution (0.1 M) for 10 min. After thorough rinsing with water, the cysteamine gold-coated glass slides were dried under nitrogen flow [53]. SLB surfaces were obtained by fusion of ~80 nm diameter liposomes having different charges on the silica sensors [41]. For details concerning the formation of lipid vesicles and the construction of supported lipid bilayers, see Supplementary Figures S1 and S2.

### 2.2. Prussian Blue Nanostructure Formation on Substrates

The growth of the PB nanostructures was performed on three substrate surfaces using the simultaneous addition method. The substrate was placed in a beaker containing 160 mL of ultrapure water. The aqueous solutions of Fe^III^Cl_3_ and Na_4_[Fe^II^(CN)]_6_ 10·H_2_O were added simultaneously using two different tubes to the beaker at a flow rate of 0.35 mL/h, using an automatic peristaltic pump REGLO (ISMATEC). The different concentrations of Fe^III^Cl_3_ and Na_4_[Fe^II^(CN)]_6_ 10·H_2_O solutions used in this work were 2, 4, and 10 mM. To maintain the initial volume constant, a tube was used to extract the solution with a flow rate of 0.7 mL/h. The mixture was magnetically stirred at 700 rpm and maintained at 25 °C during the reaction. The substrate was then thoroughly rinsed and sonicated for 10 min with ultrapure water, and finally dried under nitrogen gas. The reactant concentrations and reaction time for each surface are summarized in the Table 1.

### 2.3. Characterization of the Surface

AFM images of PB growth were acquired in an air environment using a commercial Multimode Nanoscope III AFM (Veeco, Santa Barbara, CA, USA) equipped with a J scanner, in Amplitude Modulation AFM mode (AM-AFM) [53,54,55]. We used OTESPA-R3 Olympus AC160TS-R3 cantilevers (Bruker France, Wissembourg, France) with nominal stiffness = 26 N/m and resonance frequency = 300 kHz. AFM images were collected at 512 × 512 pixels, with scan size of 5 μm × 5 μm and 1 μm × 1 μm, and with a speed of one line per second. Images, data and profile analysis, flattening as well as statistical evaluation of film coverage and roughness were carried out using Gwyddion Software [56]. Infrared (IR) spectra were recorded using a Spectrum Two FT-IR Spectrophotometer in attenuated total reflection mode. Structural properties were studied by X-ray diffractometry (XRD) in the 2θ interval 0−50 °C at room temperature (Malvern Panalytical X-Pert PRO, Palaiseau, France).

### 2.4. Photothermal Experiences

Photothermal experiences were realized on sample 2c. We used a 808 nm laser (Kamax society, Limoges, France) with an adjustable power from 0 to 2.58 W/cm^2^. The laser spot surface was 0.32 cm^2^. The temperature of the surface was measured using an OPTRIS PI 450 thermal camera (Media Mesures, Bouc Bel Air, France). The sample was deposed on a polystyrene surface in contact with air. To avoid the infrared mirror problem for the temperature measurement of the surface, a transparent surface at 808 nm but opaque in the camera infrared range was placed on the surface (0.1 mm piece of adhesive tape). Without the additional surface, no temperature measurement can be realized.

### 2.5. Photothermal Simulations

Photothermal simulations of PB surfaces were realized using COMSOL software. The heat equation was solved simultaneously with the simplified Navier–Stokes equation to take into account the heat propagation and the convection term present for the air. The model consisted of a glass layer, with a surface heat source to modelize the heat produced by the laser absorption of the PB layer, deposed on a polystyrene support with a 0.1 mm adhesive tape on its surface in contact with the air.

## 3. Results and Discussion

In this study, we investigated the controlled growth of PB nanostructures by using a simple “one-pot” approach involving the simultaneous addition of precursors by using three different planar surfaces: bare gold, cysteamine-functionalized gold, and glass-supported lipid bilayers.

**Figure 1:**
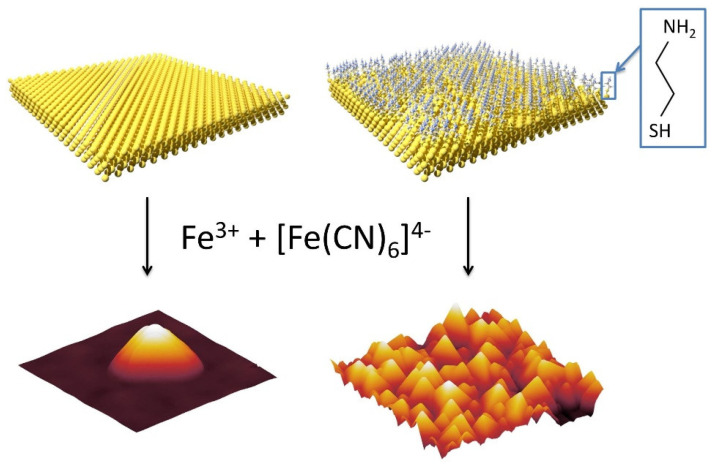
Schematic representation of the used approach for the growth of the PB nanostructures on the bare gold surface (**left**) and functionalized by the cysteamine gold surface (**right**).

First, we considered the bare gold-coated glass surface (Figure 1, left) because Au^0^ presents an affinity to cyanides permitting the direct linkage of cyanometallate precursors or PB nanoparticles, as it has previously been demonstrated in the case of the epitaxial growth of the PB film on the Au(110) single crystalline surface [49] or in the case of Au@PBA core-shell nano-objects [46]. Second, we performed the functionalization of the gold-coated glass with the conventional cysteamine molecule accordingly to a previously reported method in the literature [53,57], in order to investigate the impact of the functionalization on the homogeneity, size, and morphology of the PB nano-objects (Figure 1, right). Indeed, the free NH_2_ group of cysteamine may be used as a nucleation site for the formation of the PB nanoparticles [53].

### 3.1. Growth of PB Nanostructures on the Gold Surface

PB nanostructures were grown by the simultaneous automated addition of Fe^III^Cl_3_ and Na_4_[Fe^II^(CN)]_6_ 10·H_2_O aqueous solutions; the whole volume of the reaction media was kept constant by the concomitant automated withdrawl of the corresponding added volume. (Supplementary Figure S3). The stirring rate, temperature, and time of the reaction were kept constant, while the reactant concentrations were varied ([FeCl_3_] and [Na_4_[Fe(CN)]_6_ 10·H_2_O at 2, 4, and 10 mM). After the reaction, the substrate was rinsed and dried prior to any characterization. Thus, two series of PB-nanostructured surfaces were obtained: PB/bare gold (1a–c) and PB/cysteamine functionalized gold (2a–c), where different concentrations of precursors correspond to different surface samples: 2 mM for (a), 4 mM for (b), and 10 mM for (c)). The IR spectra confirm the formation of the PB network in all cases.

**Figure 2:**
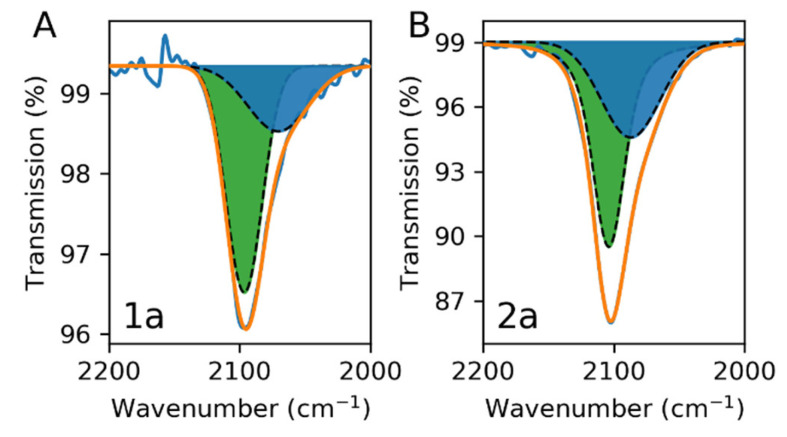
ATR-IR spectra in the 2000–2200 cm^−1^ window for samples 1a (**A**) and 2a (**B**).

The ATR-IR spectra of selected samples 1a and 2a, shown in Figure 2, exhibit a unique band at around 2100 cm^−1^ (at 2097 and 2102 cm^−1^ for 1a and 2a, respectively) with a shoulder at lower frequency. This band can be deconvoluted with two major contributions at 2080 cm^−1^ and 2100 cm^−1^, which may be attributed to the stretching vibrations of the coordinated Fe^II^-C≡N-Fe^III^ cyanides in the PB network in its non-cationic (Fe^III^[Fe^II^(CN)_6_]_3/4_▯_1/4_·*n*H_2_O) and cationic (A_1−x_Fe^III^[Fe^II^(CN)_6_]_1−x/4_▯_x/4_·*n*H_2_O) forms, respectively, as previously reported by Hamnett, et al. [58] XRD experiments were also performed to characterize the crystallinity of our samples. However, due to the low amount of material deposited on the gold surfaces, the XRD signal of PB was not visible for almost all samples. Only sample 2c, which was synthesised with high concentrations of precursors, showed PB XRD peaks. In XRD patterns of the PB nanoparticles, the (200), (400), and (220) peaks were visible, and the (222) peak was not visible; examples of such kinds of patterns are given by Fumiyuki Shiba [59,60]. Sample 2c had intense reflections associated with the (200) and (400) planes. The strong intensity of these two peaks demonstrates the cubic structure of sample 2c growth along the (200) crystal axis (Supplementary Figure S4) with a typical *fcc* structure with a lattice parameter *a* = 10.13 Å.

**Figure 3:**
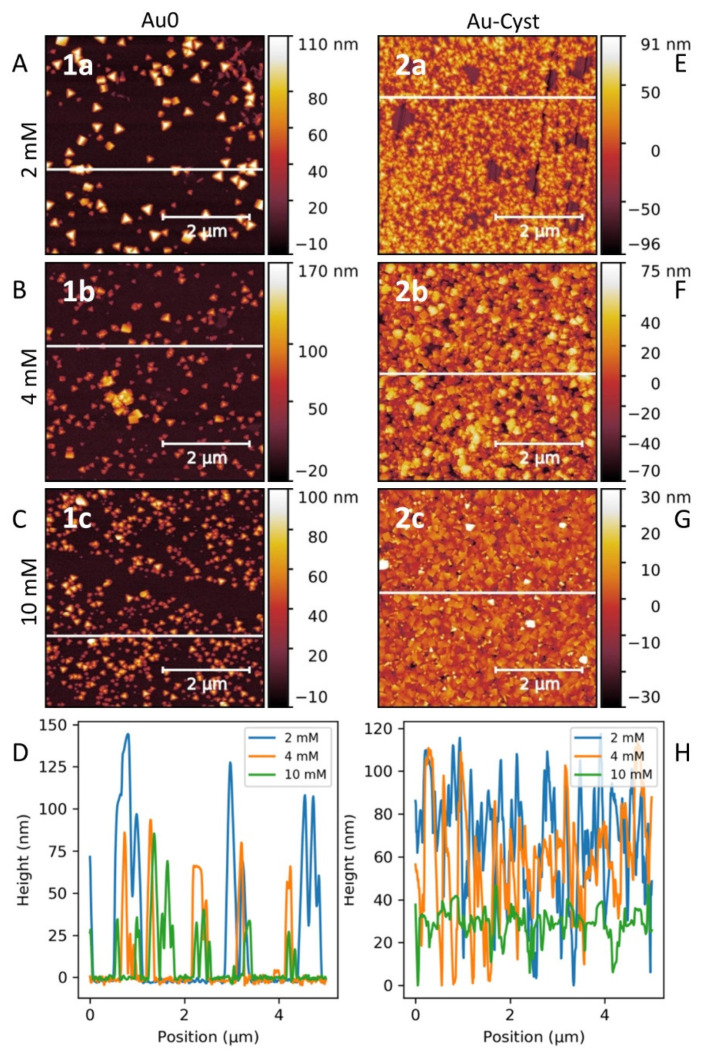
AFM images (**A**–**C**,**E**–**G**) for samples 1a–c–2a–c corresponding to the growth on bare gold and cysteamine-functionalized gold surfaces with different precursors at concentrations of 2 mM (1a, 2a), 4 mM (1b, 2b), and 10 mM (1c, 2c) and the corresponding height profiles (**D,H**).

AFM is a powerful technique for surface characterization. Indeed, the size and the morphology of the formed PB nano-objects, as well as the homogeneity of the PB surface covering have been investigated by this technique. Figure 3A–D present AFM images of PB nanostructures on bare Au surface 1a–c showing, for PB nano-objects, unusual well-formed isolated triangular nanopyramids (nano-tetraedron) with heights in the range 25–125 nm. The pyramidal morphology is the same for the three samples, but the size of these nano-objects decreases and their amount deposited on the surfaces increases as the concentration of the added precursors increases. The size reduction is clearly visible on the height profiles of the three images (Figure 3D). This fact may be explained regarding the nucleation/surface growing mechanisms, where the nucleation will be favoured in the case of higher precursor concentrations.

Besides the original pyramidal shape and different size, the inhomogeneity of the surface coverage may also be noted. It is due to the low probability to create a nucleation point of PB on an Au^0^ surface. The probable scenario to explain the creation of nucleation points consists of a local modification of the Au surface with a [Fe^II^(CN)_6_]^3−^ moiety, due to the chemical affinity between the gold and cyanides [61]. The variation of the reaction kinetic produced in our case by a variation of the reactant concentrations has a visible impact on the nucleation-growth process. AFM images (Figure 3A–C) and the height profile (Figure 3D) show that nucleation is favoured by the increase of the addition rate. Conversely, a reduction of the addition rate favours the process of the nano-object growth, leading to sparser structures, with larger perimeters. It must be noted that irrespective of the addition rate, the pyramid shape remains unchanged.

PB growth on cysteamine-functionalized gold surface indicates two important effects: (i) the almost total and homogeneous covering of the gold surface with the PB nano-objects and (ii) the observed impact of the reactants′ concentration on the shape of the obtained PB nanostructures 2a–c. At the lowest concentration, the growth of triangular nanopyramids is predominant, while at higher concentrations, cubic shaped PB nano-objects deposited on the surface were formed (Figure 3E–G). The corresponding AFM height profiles (Figure 3H) show that at the highest concentration (2c), the roughness of the film was reduced to around 20 nm. Increasing the concentration of the reactants, which can be viewed as an augmentation of the addition rate, will favour the nucleation process. This implies a larger amount of nucleation sites resulting in a higher coverage of the surface. As expected, the cysteamine functionalization of the gold surface permits the optimal and controlled PB surface deposition, permitting the nanocrystalline film growth thanks to coordination of free NH_2_ groups to the iron ions situated on the surface of the PB nano-objects.

Due to its cubic crystal structure, PB and its analogues above ca. 10 nm usually form cubic nanoparticles [14,15,62]. Thus, the intuitive expectation should be the nucleation and growth process of nanocubes on a surface, as was observed in the literature for the synthesis of PB by the layer-by-layer method [39]. This shape was obtained for samples 2b,c, on cysteamine-functionalized gold surfaces at a high concentration of reactants. For the samples on pure gold 1 or cysteamine-functionalized gold at low concentration 2a, unusual PB triangular nanopyramids were obtained.

**Figure 4:**
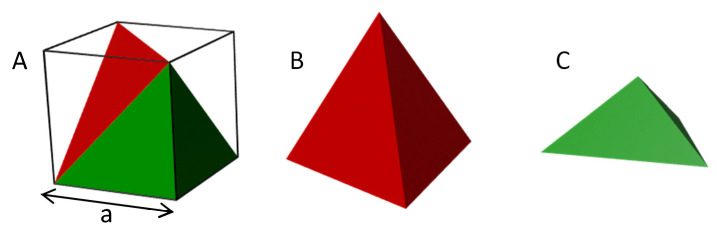
(**A**) Representation of a cube with its two triangular pyramids (tetrahedron) along the long diagonal axis. (**B**) Regular triangular pyramid extracted from the cube. (**C**) Non-regular triangular pyramid extracted from the cube.

This latter morphology has been reported in the literature only once, in the case of epitaxial PB growth on single crystal Au(110) support [49]. Taking into account that our nanopyramids and nanocubes present almost the same size, we can conclude that this effect is not linked to the cubic crystal structure of PB, but only depends on the concentration and the surface nature. Visibly, under certain experimental conditions, the presence of a planar surface seems to disturb the nucleation-growth process, preventing the formation of the cubic structure. Figure 4A shows a scheme of a cube cut into two triangular pyramids oriented along the long cube′s diagonal: one is a regular tetrahedron with four identical faces (Figure 4B) and the other is a non-regular tetrahedron with a base and three identical faces (Figure 4C). If we make the analogy of this cube with a PB crystal cell, all the faces of the regular tetrahedron are all oriented in the (222) plans. For the non-regular tetrahedron, if the base is orientated in a (222) plan, the three other faces are oriented in the (200) plans. The height of the experimental tetrahedrons measured by AFM compared to the perimeter of their bases indicates that the tetrahedron formed at the surface corresponds to the non-regular one shown on Figure 4C with a [222] epitaxial growth. The XRD experiments could provide information about the crystal orientation to confirm directly the relevance of this model, but, unfortunately, the pyramid density on the sample is too low for this kind of experiment. A simple thermodynamical approach can be used to understand why the tetrahedral shape with the [222] epitaxial growth is preferable in some cases to a cubic shape with a [200] one (see ESI for calculations). For the sake of comparison, we took as model shapes a tetrahedron and a cube with the same volume, since they contain the same quantity of matter. Then, the only differences in the Gibbs free energies between them is the surface energies. The calculation demonstrated that the energy of the interface between PB and gold is the only surface energy playing a real role in the Gibbs free energy. The surface energy for the PB/gold interface for nanocubes is smaller than in the case of nanotetrahedrons, and the crystal faces at the interface with gold are not the same: face (100) and face (111) for nanocubes and nanotetrahedrons, respectively. Then, a better fit between the gold surface and the PB tetrahedron cell parameter can reduce the PB/gold surface energy. Gold and PB adopt a face-centred-cubic and cubic NaCl-type structures with cell parameters of 4.08 Å and 10.15 Å, respectively. The lowest misfit between the gold and PB cell parameter is obtained for a (100) Au and a (222) PB plan. The obtained distance between three gold atoms along the (100) plan diagonal ([110] axis) is 17.03 Å, which has good adequacy with the distance of 17.58 Å between two Fe^II^-Fe^II^ in a (222) PB plan. Therefore, the variation of the concentration of reactants can also be seen as a variation of the synthesis kinetic. For low kinetics of the reaction, the shape of the nanostructures tends to favour the thermodynamical equilibrium and minimize the interfacial energy between the substrate and the PB; then, the triangular pyramid is obtained. For higher kinetics, the system can be trapped in a metastable state, and cubic nanostructures are obtained.

**Figure 5:**
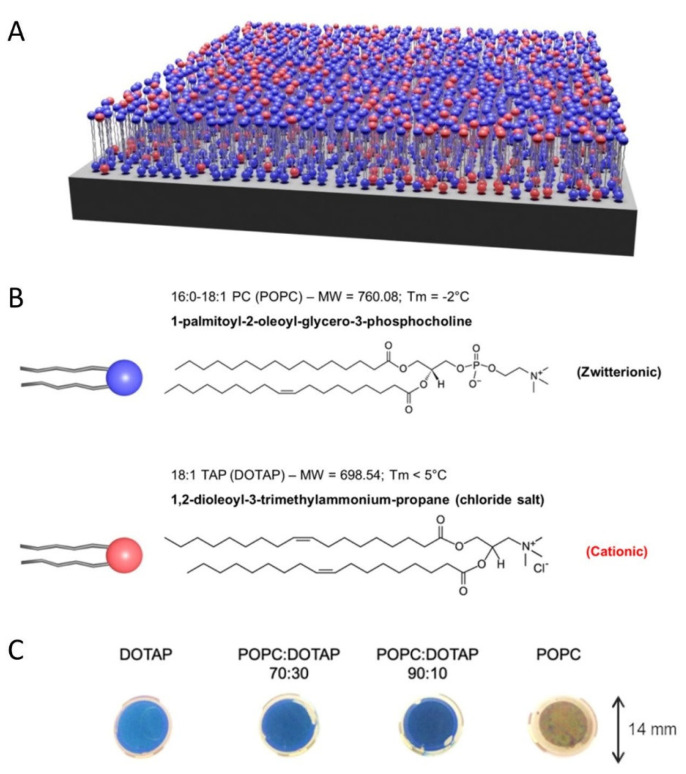
Schematic representation of the used SLB surfaces, example with 70% of POPC and 30% of DOTAP lipid bilayers (**A**) and chemical formula of the corresponding lipids POPC and DOTAP (**B**). Photographs of QCDM sensors after PB deposition on top of the SLB/SiO_2_ surface (**C**).

**Figure 6:**
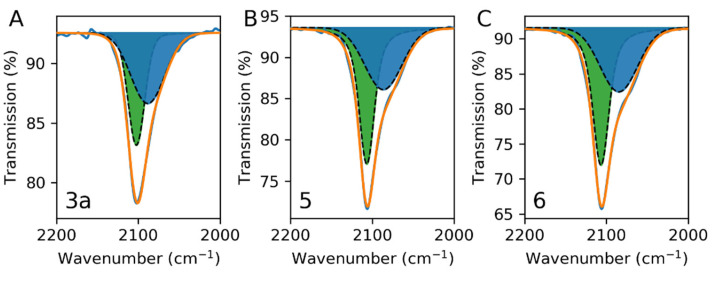
ATR-IR spectra in the 2000–2200 cm^−1^ window for samples 3a (**A**), 5 (**B**), and 6 (**C**).

SLBs are cell-membrane-mimicking versatile assemblies in terms of surface charge that can be formed on solid surfaces including silica, allowing modulation of the electrostatic interactions with PB. Liposomes with different ratios of 1-palmitoyl-2-oleoyl-sn-glycero-3-phosphocholine (POPC) (zwitterionic, neutral charge) and 1,2-dioleoyl-3-(trimethylammonium) propane (DOTAP) (cationic, positive charge) were used here to form SLB surfaces (Figure 5). POPC liposomes exhibited a Zeta potential of −2.0 ± 0.1 mV. Increasing the DOTAP amount in the POPC/DOTAP mixtures allowed the modification of the surface charge of the formed liposomes: liposomes with a POPC:DOTAP ratio of 90:10, 70:30, and 0:100 exhibited Zeta potentials of +14.4 ± 1.1, +22.4 ± 0.7 mV and +37 ± 1.3 mV, respectively. Indeed, different POPC/DOTAP ratios should impact the growth of the PB nanostructures taking into account that they possess a negative surface charge. The SLB on silica surfaces was obtained after liposome fusion and was characterised by QCM-D measurements (for experimental details see Supplementary Materials, Supplementary Figure S2). PB nanostructures were grown using the one-pot method with different concentrations of [FeCl_3_] and [Na_4_[Fe(CN)]_6_ 10·H_2_O precursors for 15 h, previously described in the case of the gold surfaces (Supplementary Figure S3). Thus, a series of PB/SLB samples were obtained and characterized: PB/100%POPC (3a–c), PB/ POPC90%-DOTAP10% (4a–e), PB/POPC70%-DOTAP30% (5), and PB/100%DOTAP (6) (2 mM for (a), 4 mM for (b), and 10 mM for (c)). Moreover, the impact of the reaction time was investigated. For this, the reaction time of 15 h for sample PB/ POPC90%-DOTAP10% 4a was reduced to 10 h to obtain sample (4d) and to 5 h for sample (4e). As in the case of the PB nanocrystals on the gold surface, the IR spectra of samples showed the unique characteristic band at around 2100 cm^−1^, attesting to the formation of the cyano-bridged PB network. The representative ATR-IR spectra for samples 3a, 5, and 6 show the bands at 2102, 2106, and 2106 cm^−1^, respectively (Figure 6). Each of them can be deconvoluted with two components at 2080 cm^−1^ and 2100 cm^−1^ attributed to the presence of non-cationic and cationic PB forms, respectively, as previously reported [58].

### 3.2. Growth of PB Nanostructures on Suppoted Lipid Bilayers

**Figure 7:**
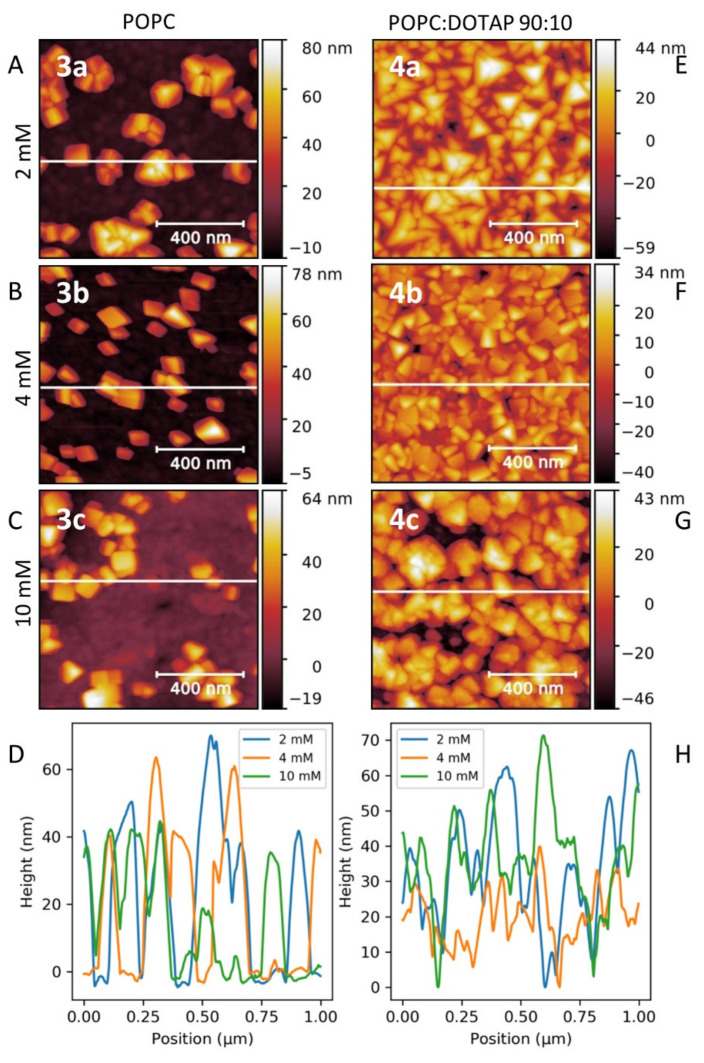
AFM images (**A**–**C**,**E**–**G**) for samples 3a–c and 4a–c corresponding to the growth on POPC (3a–c) and POPC90%-: DOTAP10% (4a–c) lipid bilayers with increasing reactant concentration of 2 mM (3a, 4a), 4 mM (3b, 4b), and 10 mM (3c, 4c) and the corresponding height profiles (**D**,**H**).

Figure 7 shows the AFM images for samples 3a–c and 4a–c. For the PB structures grown on a pure POPC bilayer (neutral charge), relatively large isolated nanocrystals without a clearly distinguished shape and an average height of 45 ± 15 nm were formed at a low concentration (Figure 7A–D). Their size decreased as the concentration of precursors increased. When DOTAP was introduced at 10% in the lipid bilayer, inducing a change in the surface charge from neutral to positive one (samples 4a–c), the formation of a dense film of the PB nanopyramids with the height profile indicating a roughness of the PB layer of around 60 nm with a homogenous surface coverage was observed at a low concentration. At higher concentration (Figure 7F,G), similar results to the cysteamine-functionalized gold surfaces were observed. At a concentration of 4 mM of precursors, a mix between nanopyramids and cubes was obtained, and at a concentration of 10 mM of precursors, the surface appeared to be composed of a structuration of smaller objects. As in the case of the functionalised Au surface, the morphology and the size of the nano-objects depend on the precursors’ concentration.

**Figure 8:**
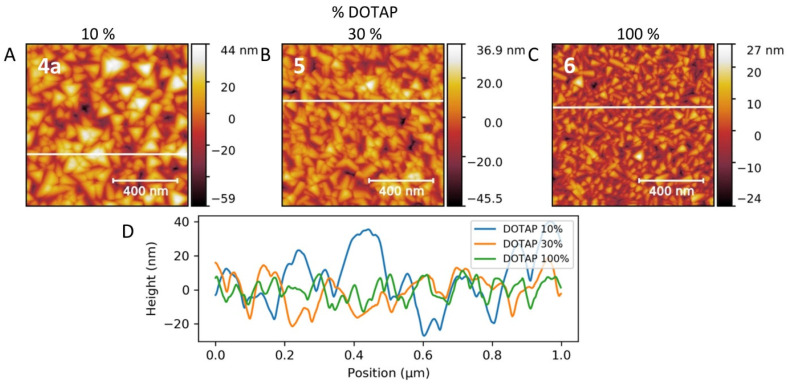
AFM images of samples 4a (**A**), 5 (**B**), and 6 (**C**) corresponding to the growth on POPC:DOTAP SLB for 10%, 30%, and 100% of DOTAP, respectively; (**D**) Height profiles for the three samples.

Moreover, when the positive charge on the surface was increased to + 22.4 ± 0.7 mV and + 37 ± 1.3 mV by increasing of the DOTAP percentage in the lipid bilayers (sample 5 and 6, respectively) (Figure 8), the size of the nanopyramids progressively decreased (the roughness decreased from 50 nm to 20 nm for 6 at the same precursor concentration of 2 mM). These results show a correlation between the surface density and the size of PB nano-structures and surface charge in the SLB. This can be explained by the ability of negatively charged PB structures to interact with neutral or positively charged surfaces of the SLB through the electrostatic interactions. The modification of the surface charge enables us to control the nucleation-growth process. When the density of the charge decreases, the number of nuclei is reduced, favouring the growth process. By contrast, when the density of charge increases, the nucleation process is favoured, leading to smaller nano-structures.

**Figure 9:**
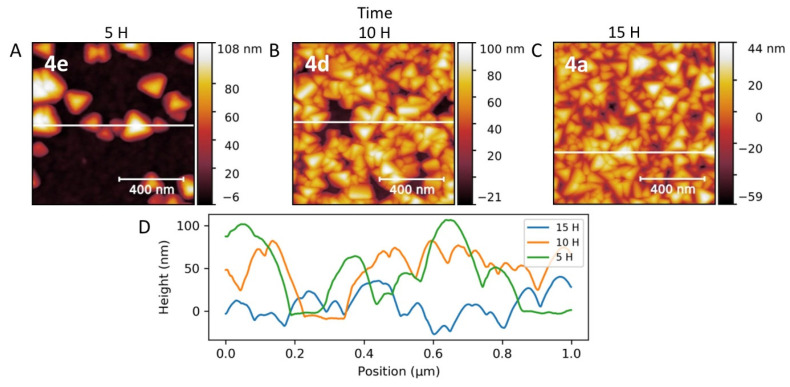
AFM images of samples 4a (**C**), 4d (**B**), and 4e (**A**) obtained by modulation of the reaction time for 15 h, 10 h, and 5 h, respectively; (**D**) Height profiles for the three samples.

To deepen the comprehension of the nucleation-growth process on the lipid bilayer surface, we decided to explore the impact of the PB deposition times by keeping the same experimental conditions as for 4a (10% of DOTAP lipid bilayers, 2 mM for the reactants). As can be seen from AFM images (Figure 9), the surface coverage by the nanopyramids progressively increased as the reaction time increased from 5 to 15 h. The nanostructure is composed of an entanglement of PB nanopyramids, which leads to a surface roughness of about 50 nm. The AFM image for a synthesis of 5 h shows isolated pyramids having a size between 20 nm and 100 nm. After 10 h, the surface coverage was almost complete with nanostructures presenting the size comparable to the one obtained at 5 h. Thus, a continuous nucleation occurred during 15 h of reaction until total coverage of the surface by the PB nanostructures. Each nucleus grew until a critical size (in this case around 100 nm), which is defined by the experimental conditions (nature of the initial surface, reactant concentrations, temperature, initial ultra-pure water volume, …).

Finally, a similar mechanism involving the direct PB growth in the case of our “one-pot” approach may be proposed for all used surfaces. It consists of (i) the formation of the nucleation site on the surface through either the direct coordination or the electrostatic linkage of [Fe^II^(CN)_6_]^4−^ on bare Au surfaces, NH_2_ groups of cysteamine or lipid bilayers of the surfaces; (ii) nuclei growth until the optimal size defined by the concentration of the reactants; (iii) the nucleation/growth process continues over time until the optimal coverage of the surface depending on the surface functionalization Note that in all cases, the blue colour of the solution at the end of the synthesis indicates the presence of PB nanoparticles. Otherwise, the possibility of an alternative mechanism consisting of the formation of the PB nanoparticles in solution with their following deposition on the surface seems to be less probable. First, we have shown in our previous work [53], that the deposition of the cubic K^+^/Ni^2+^/[Fe(CN)_6_]^3−^ PBA nanoparticles on a cysteamine-gold surface leads to a regular distribution of isolated cubic nano-objects with the size corresponding to the one of the initial nanoparticles. Second, this alternative mechanism could not explain the difference in the size and morphology of the obtained nano-objects. Besides, the repulsive interaction between the negatively charged PB nanoparticles themselves does not promote the formation of a continuous film on the surface.

### 3.3. Photothermal Properties of PB Nanostructures

Photothermal properties of PB nano-objects attract a particular interest in view of their use as efficient and photostable therapeutic agents for cancer and bacteria treatment, [63] but only recent works were devoted to the PTT investigations involving PB nanoparticles deposited on the surface [44,45]. In this work, we investigated the photothermal properties of PB nanoparticles grown on the surface by combining experimental results with theoretical simulations. Sample 2c involving PB nanoparticles deposited on and functionalised with the cysteamine Au surface was chosen because (i) the PB nanoparticles present homogeneity in size and shape, (ii) they are homogeneously deposited on the Au surface, which is completely covered by the nanoparticles, (iii) the nanoparticles are covalently attached to the Au surface and the heating provided during photothermal experiments cannot remove them, (iv) the gold substrate does not produce heating under laser light irradiation (see Supplementary Figure S5 in ESI).

**Figure 10:**
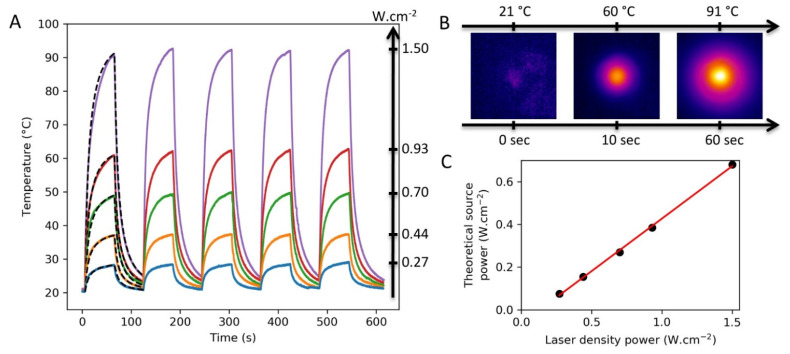
(**A**) Experimental and simulation results of PB surface laser heating (λ_ir_ = 808 nm). The plain lines represent the experimental PTT data obtained with sample 2c for different values of the laser power density represented by the left arrow. The dashed lines represent the simulation results with a density power source of 0.075, 0.155, 0.270, 0.385, and 0.680 W.cm^−2^, which were adjusted using the 0.27, 0.44, 0.70, 0.93, and 1.50 W.cm^−2^ experimental curves, respectively; (**B**) Thermal camera images of sample 2c under laser irradiation of 1.50 W.cm^−2^; (**C**) Theoretical density power source obtained from the simulations as a function of the laser density power; the plain line is a linear regression fit.

Figure 10A shows experimental results of surface laser heating of sample 2c performed by using different laser density source powers ranging from 0.27 to 1.50 W.cm^−2^. As expected, an important temperature increase depending on the laser power source was observed under irradiation at 808 nm. For instance, the local surface temperature increased to 90 °C in only 1 min with a laser power of 1.50 W.cm^−2^, demonstrating indeed a great potential of these surfaces for various applications. Figure 10B shows experimental thermal images of sample 2c irradiated with a laser density power of 1.50 W.cm^−2^ at three different times, from 21 °C to 91 °C. The stability of these PB nano-object-covered surfaces under irradiation has been proved by performing several heating cycles. Importantly, the AFM measurements after PTT experiments indicate that no modifications of size and shape of the PB nano-objects on the surface were observed.

The observed temperature difference (ΔT) is coherent with the photo-thermal response of an NP monolayer [44,64,65]. A deeper comparison can be done between our work and the work of Dacarro, et al. [45]. For a monolayer of PB NPs of 29 ± 8 nm irradiated at 808 nm under a laser power of 0.47 W/cm^2^ for 60 s, an increase in the surface temperature of 10 °C was obtained. In this work, we used similar conditions (same wavelength and irradiation time, and a power of 0.44 W/cm^2^), and an increase in the surface temperature of 17 °C was obtained. The difference between the two results can be associated with a higher density of PB nano-objects on the surface obtained by our new deposition method.

A theoretical modelling of photothermal properties, using COMSOL software, was realized to describe the experimental data (dashed lines of Figure 10A) and extract the theoretical source power. The model takes into account the geometry of the problem and its different components (polystyrene, glass layer, adhesive, and air). The PB layer was modelized by a heat density power source located on the surface of the glass layer. For each experimental curve, the density power source, produced by the PB layer, was adjusted to obtain good adequacy between experience and theory. Figure 10C shows the theoretical density source power obtained from the simulations as a function of the laser density power. The dependency between both parameters is linear (red curve) with a slope of 0.49 ± 0.01, which indicates a conversion of the laser power of almost 50% in power source heat. To the best of our knowledge, this light-to-heat conversion efficiency was calculated for PB nanoparticles organised on the surface for the first time.

## 4. Conclusions

In summary, we developed a simple and efficient “one-pot” approach to the controlled chemical growth of PB nanostructures on three different surfaces, bare gold, amino-gold, and SLB surfaces. We have demonstrated that this process leads to the formation of well-defined isolated PB nanopyramids epitaxially grown on the bare Au surfaces. Meanwhile, the optimal coverage, regularity in the PB nanostructures, and shape control were obtained with the functionalized cysteamine gold surfaces by modifying the precursors’ concentrations. This approach has been transposed to the direct construction of PB on planar SLBs, which to our knowledge has not been performed until now. By modulating the cationic lipid concentration in the lipidic surfaces, we achieved fine control of the PB coverage and demonstrated the presence of favorable electrostatic interactions between the negatively charged PB nano-objects and positively charged lipids of the SLB.

The photothermal properties were investigated by experimental methods combined with theoretical modeling. An important temperature increase, highly dependent on the laser power density, as well as high photothermal stability of PB nano-object-covered surfaces was observed, confirming their great potential as photothermal agents.

This approach opens a promising way towards the growth of regular PB films on different kinds of substrates and investigation of their photothermal properties, which could not be achieved by the conventional electrochemical deposition method due to the fast crystallization rate of PB [29]. This simultaneous addition method for the growth of PB nanostructures with Au or SLB surfaces is low-cost, automatically controlled, and can be easily scaled up for the nano-construction of biosensor, memory, micro/nano electronics devices, and antibacterial surfaces, etc. [66,67,68].

## Figures and Tables

**Table 1:** Experimental conditions of PB growth on different surfaces.

Samples	Surfaces	Reactant Concentrations (mM)	Reaction Time (h)
1a	Gold	2	15
1b	Gold	4	
1c	Gold	10	
2a	Amino-Gold	2	
2b	Amino-Gold	4	
2c	Amino-Gold	10	
3a	POPC100% SLB	2	
3b	POPC100% SLB	4	
3c	POPC100% SLB	10	
4a	POPC90%: DOTAP10% SLB	2	
4b	POPC90%: DOTAP10% SLB	4	
4c	POPC90%: DOTAP10% SLB	10	
4d	POPC90%: DOTAP10% SLB	2	10
4e	POPC90%: DOTAP10% SLB	2	5
5	POPC70%: DOTAP30% SLB	2	15
6	DOTAP100% SLB	2	

## Data Availability

The data are available within the manuscript and the corresponding supporting information file.

## Supplementary Materials

The following are available online at https://www.mdpi.com/article/10.3390/nano11071749/s1, Supplementary Figure S1: Characteristics of POPC and DOTAP lipids, Supplementary Figure S2: QCM-D monitoring of different charged SLB formations on SiO_2_ surfaces presented at the 7th harmonic, Supplementary Figure S3: Scheme of the experimental process for the deposition on the surface, Supplementary Figure S4: X-Ray Diffractograms for sample 2c. The symbol * represents the XRD gold peak of the substrate, Table S1: Physicochemical characterization of lipid vesicles composed of different proportions of POPC and DOTAP in HBS buffer (20 mM Hepes, 150 mM NaCl, pH 7.4). Supplementary Figure S5: Experimental results of sample 2c and gold substrate surface laser heating (λ = 808 nm), with a laser density power of 0.72 W cm^−2^.
