# Supplementary material for: A Novel Approach to the Facile Growth and Organization of Photothermal Prussian Blue Nanocrystals on Different Surfaces
Source: Nanomaterials (Basel). 2021 Jul 2;11(7):1749. doi: 10.3390/nano11071749 (PMC8308188; doi:10.3390/nano11071749)
Supplement: Supplementary file 1 [file nanomaterials-11-01749-s001.zip › nanomaterials-1235843-supplementary.pdf]

Supporting Information

A Novel Approach to the Facile Growth and Organization of Photothermal Prussian Blue Nanocrystals on Different Surfaces

Giang Ngo [1], Gautier Félix [1,*], Christophe Dorandeu [1], Jean-Marie Devoisselle [1], Luca Costa [2], Pierre-Emmanuel Milhiet [2], Yannick Guari [1,*], Joulia Larionova [1] and Joël Chopineau [1,*]

[1] ICGM, Univ Montpellier, CNRS, ENSCM, 34090 Montpellier, France; honggiang91@gmail.com (G.N.); christophe.dorandeu@umontpellier.fr (C.D.); jean-marie.devoisselle@umontpellier.fr (J.-M.D.); joulia.larionova@umontpellier.fr (J.L.)
[2] CBS, Univ Montpellier, CNRS, INSERM, 34090 Montpellier, France; costa@cbs.cnrs.fr (L.C.); pierre-emmanuel.milhiet@cbs.cnrs.fr (P.-E.M.)
* Correspondence: gautier.felix@umontpellier.fr (G.F.); yannick.guari@umontpellier.fr (Y.G.); joel.chopineau@enscm.fr (J.C.)

S1: Formation of lipid bilayer planar substrates

1-palmitoyl-2-oleoyl-glycero-3-phosphocholine (POPC) and 1,2-dioleoyl-3-trimethylammonium propane (DOTAP) lipids were used in different proportions to prepare liposomes having different surface charges. The molecular mass, formula, charge and Tm of these two lipids are presented in the following figure.

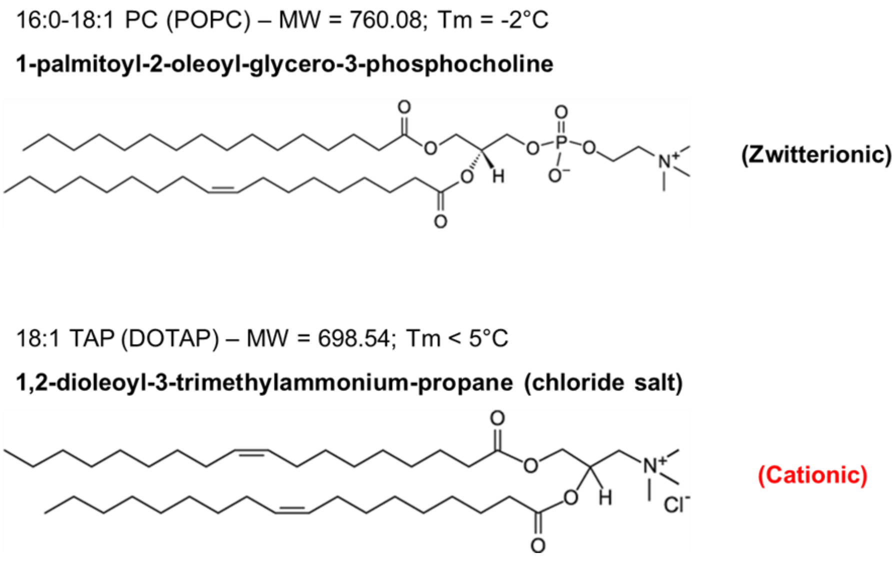

Figure S1. Characteristics of POPC and DOTAP lipids

The lipid vesicles were formed according to the Bangham method [1]. A stock solution of lipids was prepared after dissolution in chloroform at a concentration of 5 mg/mL, and stored at -20°C. A lipid film was formed from this stock solution at the bottom of a glass flask, by evaporation of solvent under nitrogen gas flow. The film was then kept under vacuum for at least 1 hour. The lipid film was rehydrated with Hepes buffer (20 mM Hepes, 150 mM NaCl, pH 7.4) to obtain a final lipid concentration of 1 mg/mL. The suspension was vortexed for 30 seconds and rested for 10 minutes, forming multilayer vesicles. This lipid suspension was then extruded back and forth manually using a Liposofast® extruder (Avestin Inc., Ottawa, Canada) using a polycarbonate filter with a porosity of 100 nm (25 times) and then one with 50 nm pores (25 times). This lipid vesicle suspension was then degassed for one minute using an ultrasonic bath (35 W, 35 kHz). The size distribution of the vesicles was estimated by dynamic light scattering (Nano ZS Malvern), whereas zeta potential was measured by electrophoretic light scattering, in the same buffer in which the liposomes were prepared.

Following this method, 4 kinds of liposomes were prepared from different lipid mixtures: pure POPC liposomes, POPC:DOTAP 90:10 (w/w) liposomes, POPC:DOTAP 70:30 (w/w) liposomes,

and pure DOTAP liposomes. The size distribution and zeta potential of the obtained liposomes are summarized in Table S1.

Table S1. Physicochemical characterization of lipid vesicles composed of different proportions of POPC and DOTAP in HBS buffer (20 mM Hepes, 150 mM NaCl, pH 7.4)

	Hydrodynamic diameter (nm)	Polydispersity index	Zeta potential (mV)
POPC	87.3 ± 1.0	0.10 ± 0.02	-2.0 ± 0.1
POPC : DOTAP 90:10	72.7 ± 0.9	0.12 ± 0.01	+14.4 ± 1.1
POPC : DOTAP 70:30	75.3 ± 0.3	0.20 ± 0.01	+22.4 ± 0.7
DOTAP	72.5 ± 0.8	0.26 ± 0.04	+37.0 ± 1.3

The formation of a supported lipid bilayer (SLB) was prepared by deposition and fusion of the vesicles on a QCM-D (Quartz crystal microbalance with dissipation) SiO_2 sensor (Biolin Scientific, Stockholm, Sweden). The operating procedure was based on the protocol of Rodahl *et al.*, [2]. The formation of the lipid bilayer was monitored using a E1 setup from Qsense or Biolin (Biolin Scientific, Stockholm, Sweden).

Before use, the SiO_2 surface of the quartz sensor was decontaminated by immersing in the solution of sodium dodecyl sulfate (SDS) 2% for 30 min, then thoroughly rinse with ultrapure water and dried under nitrogen gas. The sensor was activated by an UV-ozone treatment for 15 minutes before being mounted in the QCM-D chamber.

The initial calibration of the quartz was carried out in air. Then, the baseline was set in Hepes buffer (150 mM NaCl, 20 mM Hepes, pH 7.4) circulating by aspiration at a flow rate of 0.1 mL/min, for 10 minutes. The liposomes were then injected at a flow rate of 0.1 mL/min at a concentration of 0.1 mg/mL. The formation of lipid bilayer on SiO_2 surface was monitored in real time based on the frequency and dissipation changes. When a lipid bilayer was formed, Hepes buffer was injected to remove the liposome residues. Finally, the bilayer was kept stable in ultrapure water for the next experiments.

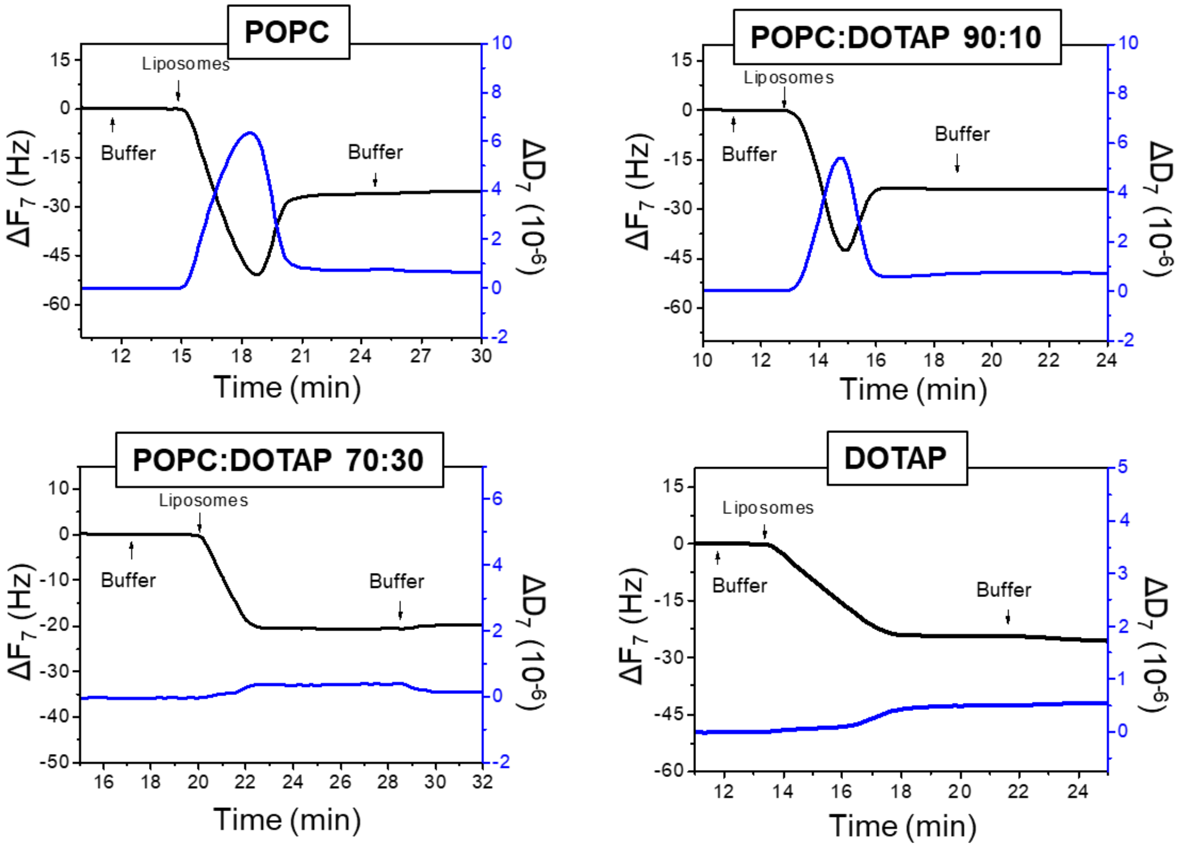

SLB composition (sample number) POPC:DOTAP	F7 shift (Hz)	D7 shift (Hz)
100:0 (3)	25 ± 1	0.8 ± 0.3
90:10 (4)	24 ± 2	1.0 ± 0.5
70:30 (5)	20 ± 2	1.0 ± 0.2
0:100 (6)	20 ± 2	0.8 ± 0.2

Figure S2. QCM-D monitoring of different charged SLB formation on SiO_2 surfaces presented at the 7th harmonic.

S2: Experimental of PB growth on flat surfaces

The growth of PB was performed on substrate surfaces using simultaneous addition method (Figure S3)

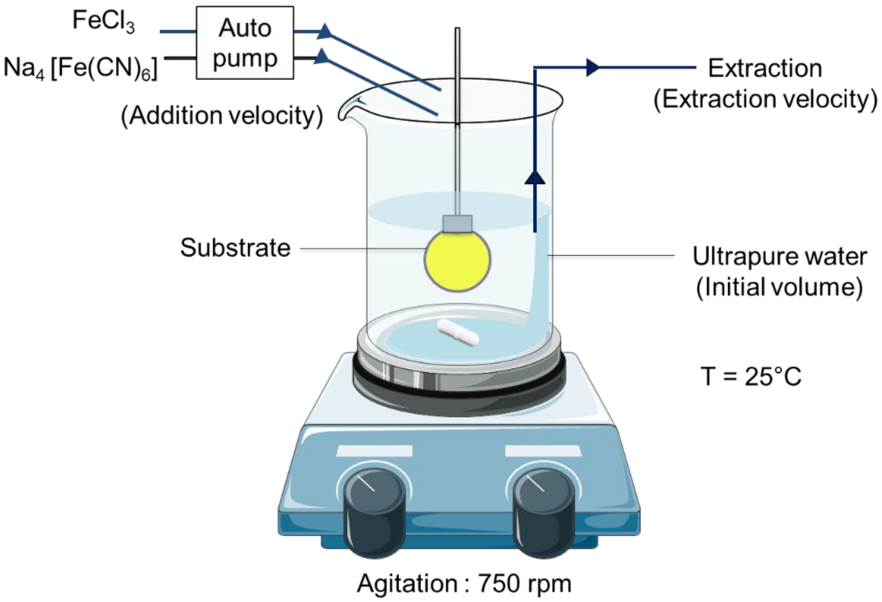

Figure S3. Scheme of the experimental process for PB deposition on the surface.

The surfaces after formation of PB assembly are shown in **Error! Reference source not found.**. We observed the blue color (characteristic of Prussian blue) stained strongly and homogenously in DOTAP, POPC : DOTAP 70:30 and POPC:DOTAP 90:10 SLB surfaces. The blue color on the sensor remained stable after all the washing steps and when sensors were stored in neutral Nitrogen gas. On the POPC sample, the blue color was also obtained but very light and not homogenous.

S3: XRD measurement

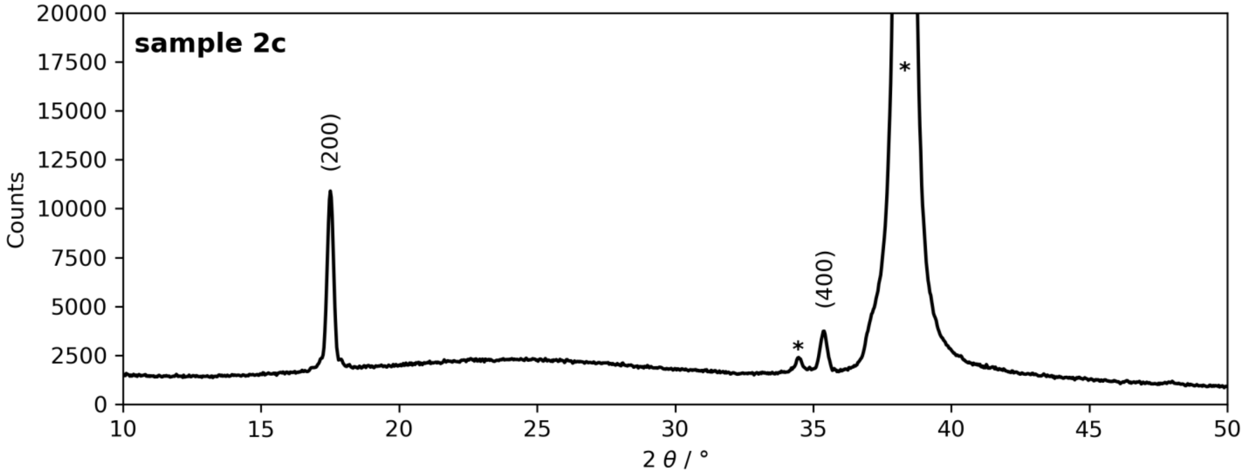

Figure S4. X-Ray Diffractograms for sample 2c. The symbol * represents the XRD gold peak of the substrate.

S4: Modelling of PB growth on a flat surface

- Tetrahedron comparison:

To explain the pyramidal shape of PB structures on the substrates we imagined how triangle-pyramids (also called tetrahedrons) could grow in a cubic pattern. Figure 4 of the article shows two ways to include tetrahedrons in a cube, following the (222) axis. If our PB structures grew along the long diagonal of the (222) axis, 2 different tetrahedrons can be obtained, a regular tetrahedron (in red on Figure 4) or a non-regular tetrahedron (in green on Figure 4). To verify this assumption, we calculated the predictive height for the red and green tetrahedrons, and compared it with experimental average height. Following the definition of a regular tetrahedron, the side a of the equilateral based triangle is equal as the third of the perimeter, and the height of the pyramids h is equal to $\sqrt{6}a/3$, for the green tetrahedron the based triangle is the same but the height of the pyramids is twice lower than the regular one $\sqrt{6}a/6$. Yet, PB crystallites average perimeters at 2, 4 and 10 mM were found to be 0.9, 0.5 and 0.3 µm, respectively. In the approximation of regular tetrahedron, to an average side of 300, 170 and 100 nm and an average height of 245, 140 and 82 nm at 2, 4 and 10 mM, respectively. However, the AFM corresponding height profiles (Figures 3A–C of the main article) show that at the lowest concentration 2 mM, PB assemblies formed polydisperse pyramids (less than 120 nm), with a sparse distribution. At higher concentration (4 mM), the pyramidal grains are smaller (around 80 nm) but their

distribution increased. The obtained pyramids are smallest (around 40 nm) while showing their highest density at 10 mM. It is interesting to note that the height obtained with the formula from a perfect tetrahedron is twice the AFM measured height. Thus, the assumption of a crystalline growth along the long diagonal of the (222) axis leading to regular tetrahedron is invalidated. This led us to consider the possibility of a PB growth following the short diagonal of this axis, leading to a non-regular flattened tetrahedron (in green on Figure S5). This second assumption is seducing as the height of this green non-regular tetrahedron included in a cube would be twice lower than the height of the red regular tetrahedron, which would fit with the experimental heights measured by AFM.

- Thermodynamical approach:

In this section we will compare the Gibbs free energies for different shapes of PB nanostructures. To be comparable we need to have the same quantity of matter, which can be approximate by an identical volume between all shapes. As the material and the volume are the same for all shapes the volume Gibbs free energy will not affect the difference in the total Gibbs free energies between the shapes. The characteristic length a_i (i = r, g, c for the red tetrahedron, the green tetrahedron and the cube, respectively) for the different shapes is defined by the width of the dark lines cube that you can see in the center of Figure S5.

The different used parameters will be:

The volume V_i, the surface at the PB/gold interface S_{ii} and the rest of surfaces S_{ri}.

The total Gibbs free energy for the shape can be written as follows:

$$G_i = V\ G_{V,i} + \sigma_{PB/Gold}\ S_{ii} + \sigma_{surface} S_{ri}$$

With $\sigma_{PB/Gold}$ and $\sigma_{surface}$ the surface energies for the PB/gold interface and the rest of the surfaces, respectively. $G_{V,i}$ represents the Gibbs free energy per volume unit.

- Red tetrahedron

$$V_r = \frac{a_r^3}{2}$$

$$S_{ir} = \sqrt{3} a_r^2$$

$$S_{rr} = 3\sqrt{3} a_r^2$$

- Green tetrahedron

$$V_g = \frac{a_g^3}{6}$$

$$S_{ig} = \sqrt{3}a_g^2$$

$$S_{rg} = \frac{3}{2}a_g^2$$

- Cube

$$V_c = a_c^3$$

$$S_{ic} = a_c^2$$

$$S_{rc} = 5a_c^2$$

- Red vs Green tetrahedrons:

If we take the same volume for the two objects, we have: $V_r = V_g \rightarrow a_r/a_g = (1/3)^{1/3}$

We have the following ratio for the PB/gold interface area: $S_{ir}/S_{ig} = a_r^2/a_g^2 = (1/3)^{2/3} \approx 0.48$

And the following ratio for the rest of surfaces: $S_{rr}/S_{rg} = 2\sqrt{3}(1/3)^{2/3} \approx 1.67$

The PB/gold interface area ratio indicates that for the same volume the green tetrahedron will have a higher interface area with the gold compare to the red tetrahedron. The other surface ratio indicates that the green tetrahedron will have a smaller surface which is in contact with the environment. Experimentally we observed green tetrahedron, which means that the PB/gold surface energy seems to be favorable and the surface energy for surface in contact with the chemical solution is unfavorable.

- Green tetrahedrons vs cubic shape

If we take the same volume for the two objects, we have: $V_c = V_g \rightarrow a_c/a_g = (1/6)^{1/3}$

We have the following ratio for the PB/gold interface area: $S_{ic}/S_{ig} = a_c^2/(a_g^2\sqrt{3}) \ = 1/\left(6^{2/3}\sqrt{3}\right) \approx 0.18$

And the following ratio for the rest of surfaces: $S_{rc}/S_{rg} = 10/\left(3 \cdot 6^{2/3}\right) \approx 1.01$

The PB/gold interface area ratio indicates that for the same volume the green tetrahedron will have a higher interface area with the gold compare to the cubic shape. The other surface ratio shows that magnitude of surfaces in contact with the environment is almost the same.

Experimentally we observed green tetrahedron, which means that the PB/gold surface energy seems to be more favorable in the case of tetrahedral shape.

REFERENCES

1. Bangham AD, Hill MW, Miller NGA. Preparation and Use of Liposomes as Models of Biological Membranes. In: *Methods in Membrane Biology*. Springer US; 1974:1-68. doi:10.1007/978-1-4615-7422-4_1

2. Rodahl M, Höök F, Fredriksson C, et al. Simultaneous frequency and dissipation factor QCM measurements of biomolecular adsorption and cell adhesion. *Faraday Discuss*. 1997;107:229-246. doi:10.1039/a703137h
